# The systemic and kidney hemodynamic response to empagliflozin, losartan and their combination varies between individuals

**DOI:** 10.1007/s40620-025-02289-3

**Published:** 2025-04-12

**Authors:** Britt Eveline Wever, Rosalie Annemien Scholtes, Charlotte Michelle Mosterd, Anne Clasien Hesp, Mark Martinus Smits, Hiddo Jan Lambers Heerspink, Daniël Henri van Raalte

**Affiliations:** 1 Diabetes Center, Department of Internal Medicine, Amsterdam University Medical Centers, Location VUMC, De Boelelaan 1117, 1081 HV Amsterdam, The Netherlands; 2 Department of Clinical Pharmacy and Pharmacology, University Medical Center Groningen, Groningen, The Netherlands

**Keywords:** Kidney Hemodynamics, SGLT2 inhibition, RAS inhibition, Diabetic Kidney disease

## Abstract

**Background:**

Renin-angiotensin system inhibitors (RASi) and sodium glucose cotransporter inhibitors (SGLT2i) are known for their kidney protective properties, but both show significant residual risk in large outcome trials. In these trials, SGLT2i were introduced on top of RASi; the individual response to each drug is currently unclear. We therefore aimed to investigate the individual reactions to the angiotensin II receptor blocker (ARB) losartan and the SGLT2i empagliflozin and their combination on measured GFR (mGFR) and systolic blood pressure (SBP).

**Methods:**

In this double-blind, randomized, 4-armed, crossover study, 24 participants received 7 days of empagliflozin 10 mg once daily, losartan 50 mg once daily, combination therapy or matching placebo. We visualized individual drug response variability. We further explored predictors of mGFR and SBP changes.

**Results:**

During empagliflozin administration, a greater than 10% reduction in mGFR was observed in 26% of participants receiving empagliflozin, in 30% of those receiving losartan, and in 39% among participants on combination therapy. In comparison, a greater than 10% reduction in SBP was observed in 35% of participants on empagliflozin, in 39% of those receiving losartan and in 43% on combination therapy. A large part of the participants who did not respond to one drug, did respond to the other drug or their combination. Monotherapy SGLT2i did not correlate with monotherapy ARB in mGFR change or SBP change.

**Conclusions:**

Our data show large individual variability in response to treatment with the ARB losartan and the SGLT2i empagliflozin. Clinicians should monitor treatment responses in patients and consider switching from one kidney protective drug to another in non-responders.

**Graphical abstract:**

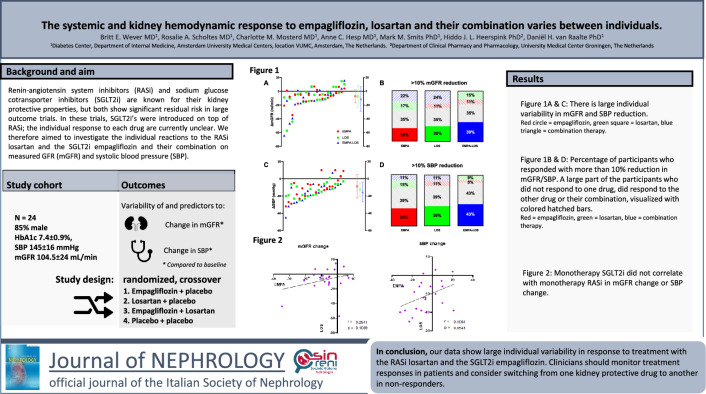

## Introduction

The incidence of diabetic kidney disease (DKD) has risen in parallel with the ongoing type 2 diabetes (T2D) epidemic. Progression of DKD is driven by various systemic (e.g. hypertension) and kidney hemodynamic factors resulting in structural damage to the glomeruli and tubular system. Regarding kidney hemodynamic changes, increased glomerular pressure, presenting as whole kidney hyperfiltration or as single nephron hyperfiltration, accelerates kidney function decline [1]. Kidney protective drugs have become available that reduce systemic and intraglomerular pressure [2]. Angiotensin II-receptor blockers (ARBs) and sodium-glucose cotransporter 2 inhibitors (SGLT2i) improve kidney outcomes in people with DKD by lowering systemic blood pressure and intra-glomerular pressure, thereby attenuating single and whole kidney hyperfiltration [3, 4]. Additionally, ARBs and SGLT2i may have suppressive effects on inflammation and fibrosis, and SGLT2i may also suppress kidney hypoxia [1, 5].

In previous trials, SGLT2i were introduced on top of ARBs. Therefore, the interaction between these drug classes or individual responses to each drug have been poorly detailed. This is of importance as all kidney outcome studies have shown significant residual risk, suggesting that not all participants responded optimally to either ARBs and/or SGLT2i [6]. The recently published RECOLAR trial provides an opportunity to address this knowledge gap [7]. In this placebo-controlled, cross-over trial, 24 participants with T2D were randomly treated with the ARB losartan, the SGLT2i empagliflozin or their combination. This study demonstrated that combination therapy reduced glomerular pressure and blood pressure to a larger extent than either of the drugs alone. As all individuals in this trial received all agents, we were able to investigate individual reactions to ARB and SGLT2i. We moreover aimed to identify individual characteristics that would predict treatment response to these drugs with respect to measured GFR (mGFR) and blood pressure responses.

## Methods

### Trial design

For this secondary analysis we used data collected in the RECOLAR trial [5, 7]. The RECOLAR trial was a phase 4, double-blind, randomized, 4-armed crossover study conducted from September 2020 to September 2021. The study was registered at ClincialTrials.gov (ID: NCT04238702). The local authorities and medical ethical review board of the VU University Medical Center (Amsterdam, The Netherlands METc no. NL71049.029.19) approved all protocol-specific documents, and all study procedures were conducted in accordance with the Declaration of Helsinki and Good Clinical Practice guidelines.

### Study population

The outpatient clinic database at the Diabetes Center Amsterdam was used to recruit participants. Participants were included if they met the following criteria: men or postmenopausal women, aged 45–75 years, body mass index (BMI) ≥ 25 kg/m2, type 2 diabetes with HbA1c levels from 6.5% to 10.5%. Participants were allowed to use metformin with or without sulphonylurea derivatives on a stable dose for ≥ 3 months prior to inclusion. Hypertension medication was actively converted 4 weeks prior to the intervention periods to alpha and/or beta blockers at a maximum tolerable dose if necessary. Exclusion criteria were unstable or rapidly progressing kidney disease, estimated glomerular filtration rate (eGFR) of less than 60 ml/min per 1.73m^2^, albumin-to-creatinine ratio > 300 mg/g, (re)current urinary tract or genital infection, diabetic ketoacidosis within 6 months before inclusion, urinary retention, use of non-steroidal anti-inflammatory drugs or diuretics that could not be discontinued 3 months before intervention periods, recent history of cardiovascular disease (i.e. < 6 months) or diagnosis of heart failure.

### Intervention

**Fig. 1:**
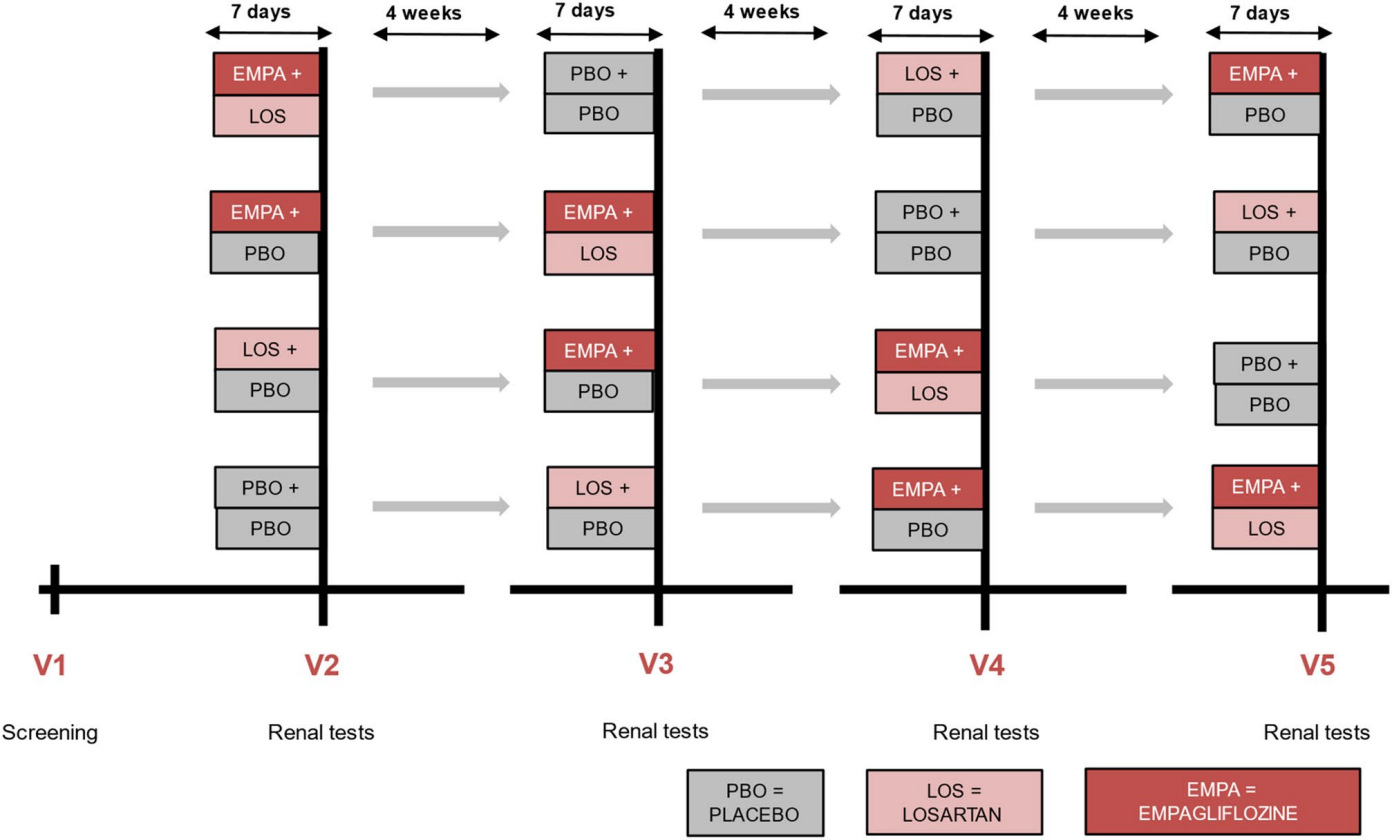
Study design

Participants were randomized to 7 days of 10 mg of empagliflozin once daily, 50 mg of losartan once daily, 10mg empagliflozin + 50mg losartan once daily or matching placebo. Between each treatment period was a washout period of 4 weeks (Fig. 1). Previous research has shown that a treatment period of 7 days is enough to observe treatment response in mGFR and SBP [5, 7–9]. In our data analysis, we could find no signs of carry-over effects, in line with the half-life of the drugs [5, 7]*.* Empagliflozin and matching placebo tablets were provided by Boehringer Ingelheim, and losartan and matching placebo were purchased from Tiofarma. Until database lock, investigators and participants remained blinded to treatment status.

### Measurements

During the 7 days of each treatment period, participants adhered to a standardized sodium chloride diet of 9–12 g per day to minimize variance in kidney physiology. Diet compliance was checked with 24-h urine sodium excretion. All measurements, including lab assessments and urine analyses, were carried out on day 7 of each treatment period, and were collected in a fasting state. SBP, diastolic blood pressure (DBP) and heart rate were determined by an automated oscillometric device (Dinamap, GE healthcare, Little Chalfont, UK). For each timepoint, the mean of three blood pressure measurements was used.

mGFR and effective renal plasma flow were determined by clearances of Iohexol and para-amminohippuric acid, respectively. Kidney hemodynamic parameters were estimated using the Gomez equations.

### Sample size

To achieve sufficient statistical power, 23 participants were needed to detect an mGFR difference of 10 ml/min (calculations were made using Stata version 11, considering (1-β) of 90% and α = 0.05 significant). To account for drop-out, 24 participants were included.

### Statistical analyses

We summarized the baseline characteristics of 24 participants as mean with standard deviation. By plotting the residuals of the outcome variables, normality of distribution was checked. Drug response variability was visualized with Waterfall plots. Since kidney protective effects include lowering of blood pressure and glomerular pressure, drug response in this analysis was defined as reduction in mGFR and SBP, respectively. A 10% reduction in mGFR and SBP was considered a significant treatment response [10, 11]. To assess the association in drug response and their predictors, correlation plots and linear regression models were used. Change in mGFR was determined by subtracting mGFR during treatment conditions from mGFR during placebo condition. Similarly, change in SBP was determined. Measurements during placebo condition were considered equal to baseline. Statistical significance was determined through Pearson’s correlation test. Data analyses and visualization were performed by using IBM SPSS Statistics 28 and GraphPad Prism 9.

## Results

We included 24 predominantly male (87.5%) and overweight (mean BMI 31.0, SD ± 3 kg/m^2^) participants with T2D with mean age 66 ± 6 years, hemoglobin A1c 7.4 ± 0.9%, treated with metformin (100%) and sulfonylurea (46%). SBP was 145 ± 16 and DBP was 86 ± 7 mmHg, while mGFR was 104.5 ± 24 mL/min. One participant was excluded from data analyses because of withdrawal during the first treatment phase.[5].

### Variability of treatment response

**Fig. 2:**
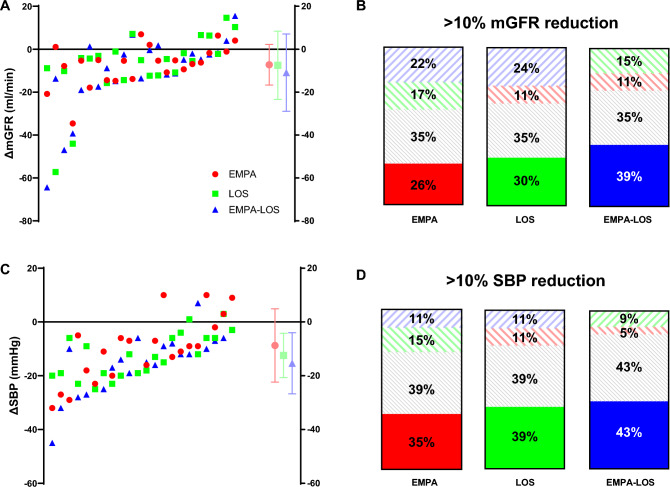
**A-D** Variability in treatment response. Figure 1A, C: Waterfall plots demonstrating mGFR/SBP changes on empagliflozin, losartan or combination therapy. Treatment response is displayed on the y-axis, participants are displayed on the x-axis. Near the right Y-axis are mean values with corresponding standard deviations. 1B, 1D: Percentage of participants who had an mGFR/SBP reduction of > 5% compared to placebo. The non-responders are displayed by the hatched bars. Grey hatched bars are participants who also did not respond to other therapies. Red hatched bars are non-responders who responded to empagliflozin monotherapy, green hatched bars are non-responders who responded to losartan monotherapy and blue hatched bars are non-responders who responded to combination therapy. *mGFR* measured glomerular filtration rate, *SBP* systolic blood pressure, *BMI* body mass index, *EMPA* empagliflozin therapy, *LOS* losartan therapy, *EMPA-LOS* combination therapy empagliflozin and losartan

Figure 2A-D demonstrates substantial variance in treatment responses between individuals. Compared to placebo, mean mGFR reduction during empagliflozin administration was 7.2 ± 9.5 mL/min, on losartan it was 7.5 ± 15.9 mL/min, and on combination treatment it was 10.9 ± 18.0 mL/min (2A). However, on the individual patient level, an mGFR reduction of 10% or more was observed in 26% of participants receiving empagliflozin, in 30% of those receiving losartan, and in 39% among participants on combination therapy (2B). Half of the participants who did not respond to one drug, did respond to the other drug or their combination, as highlighted by the colored hatched bars (Fig. 2B).

Similar to mGFR reduction, a large variation in SBP reduction was observed, with the greatest mean SBP reduction observed during combination therapy (Fig. 2C). A greater than 10% reduction in SBP was observed in 35% of participants receiving empagliflozin, in 39% of those receiving losartan, and in 43% among participants on combination therapy. Again, a significant part of non-responders did respond to other therapies (Fig. 2D, hatched bars).

### Interaction in treatment response

**Fig. 3:**
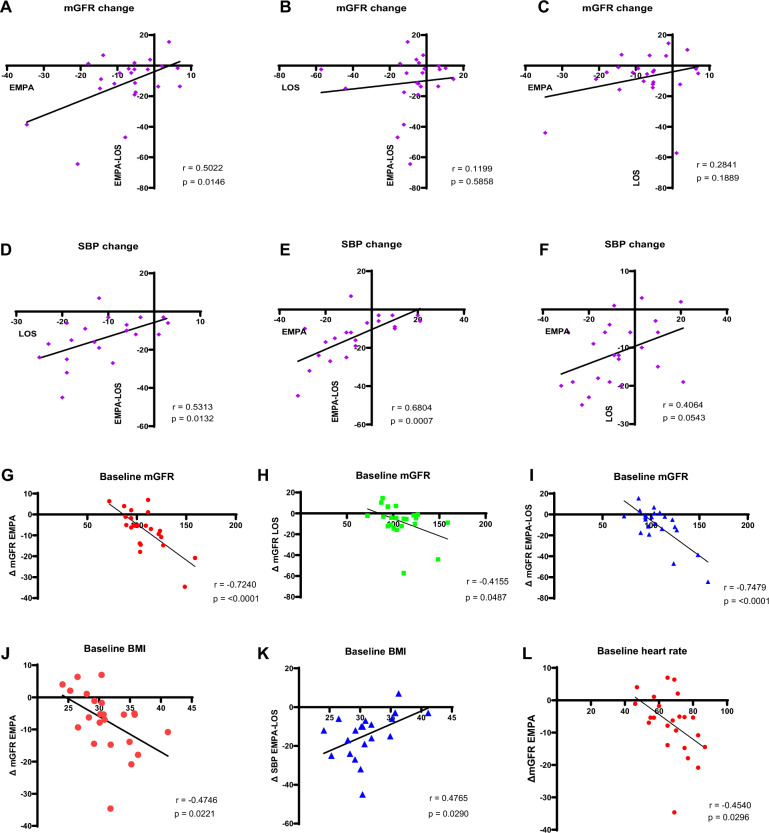
**A**–**F** Interaction in treatment response and predictors of treatment response. Displayed are correlation plots with corresponding Pearson’s correlation coefficient, p-values and linear regression lines. *P*-values < 0.05 were considered significant. **A**–**F** show interactions in treatment response. Treatment response is expressed as GFR or SBP change compared to placebo. Figures 3G–2L display baseline characteristics associated with treatment response

Change in mGFR during empagliflozin and change in mGFR during combination therapy were correlated (Fig. 3A), but this was not observed for losartan monotherapy and combination therapy (3B). Both empagliflozin and losartan monotherapy correlated to SBP changes during combination therapy (Fig. 3D, 3E). There was no correlation in treatment response between empagliflozin monotherapy and losartan monotherapy with respect to mGFR or SBP (Fig. 3C, F), although a trend was observed for SBP changes (Fig. 3F).

### Predictors of treatment response

Baseline mGFR correlated with change in mGFR for all treatment groups (Fig. 3 G, H, I), as opposed to baseline SBP, which was not correlated to treatment-induced SBP changes (data not shown). Fig. 3 J and K show that baseline BMI was negatively correlated with change in mGFR during empagliflozin treatment but positively correlated with SBP change during combination therapy. Furthermore, a significant correlation between baseline heart rate and mGFR reduction during empagliflozin was observed (3L).

No significant correlations were found between baseline urine sodium excretion, urinary volume, microalbuminuria, or other measures of kidney hemodynamic function and changes in mGFR and SBP.

## Discussion

The main findings of the current study are: 1) there is large individual variability in mGFR and SBP response to SGTL2i and ARB therapy; 2) response to one of the drugs does not predict their response to the other; 3) based on readily-available clinical parameters, no predictors of optimal response to either of the drugs could be identified.

Despite the advent of several novel kidney protective drugs, DKD remains a huge burden on patients and society. This may be reduced by matching the appropriate drug with the right patient by conducting personalized medicine. Here, we used mGFR and SBP as treatment biomarkers.

Although DKD is defined by GFR decline, the GFR reduction observed with ARBs and SGLT2i is considered beneficial. Previous analyses from the CREDENCE and RENAAL trials show that a GFR drop of more than 10% and up to 30% results in a more favorable eGFR trajectory over time compared to a GFR drop of less than 10%, irrespective of baseline eGFR [10, 11]. We speculated that participants would react with a similar GFR dip to ARB and SGLT2i, as they both lower efferent glomerular pressures [4], however this was not confirmed. Importantly, a large part of monotherapy non-responders did respond to the other therapies in terms of GFR drop*.* Thus, our data indicate that clinicians should monitor for clinical measures such as eGFR, creatinine levels, albuminuria and/or SBP after initiation of kidney protective drugs. Consequently, if patients seem to be non-responders to the drug prescribed in one or more of these clinical measures, clinicians should switch to other compounds.

Individual response with respect to SBP reduction during ARB administration did not predict response during SGLT2i. This may be an expected outcome because the underlying systemic effects driving blood pressure reductions differ somewhat for ARBs and SGLT2i [5]. ARBs reduce blood pressure by inhibiting vasoconstrictive actions of angiotensin II, and it is postulated that SGTL2i lower blood pressure via volume contraction of the interstitial volume [5].

Although individuals showed distinct responses to SGLT2i and ARBs, no clear patterns in predictive parameters were identified. The correlation between BMI and GFR change might be explained since obesity associates with hyperfiltration, which corresponds to the observation that higher baseline GFR was also associated with greater GFR reduction in all three treatment arms. The correlation between heart rate and GFR change during empagliflozin could be attributed to the suppressive effects of SGLT2i on the sympathetic nervous system [5]. Nonetheless, these correlations should be interpreted with caution. Due to detailed phenotyping and a cross-over design of the RECOLAR trial, the number of included participants was limited. Another limitation of our study is limited generalizability of the results since we mostly included White men with preserved kidney function [7]. The use of additional biomarkers, such as panels using metabolomic or proteomic stratification, could help to identify responders to treatment in future studies.

To conclude, our data show large individual variability in response to treatment with the ARB losartan and the SGLT2i empagliflozin, with individuals being non-responders to one drug and responders to the other. Clinicians should monitor treatment responses in patients, and consider switching from one kidney protective drug to another in non-responders.

## Data Availability

The data that support the findings of this study are available from Daniël van Raalte upon reasonable request.
